# Molecular Interactions From the Density Functional Theory for Chemical Reactivity: The Interaction Energy Between Two-Reagents

**DOI:** 10.3389/fchem.2022.906674

**Published:** 2022-06-13

**Authors:** Ramón Alain Miranda-Quintana, Farnaz Heidar-Zadeh, Stijn Fias, Allison E. A. Chapman, Shubin Liu, Christophe Morell, Tatiana Gómez, Carlos Cárdenas, Paul W. Ayers

**Affiliations:** ^1^ Department of Chemistry and Quantum Theory Project, University of Florida, Gainesville, FL, United States; ^2^ Department of Chemistry, Queen’s University, Kingston, ON, Canada; ^3^ Department of Chemistry and Chemical Biology, McMaster University, Hamilton, ON, Canada; ^4^ Research Computing Center, University of North Carolina, Chapel Hill, NC, United States; ^5^ Université de Lyon, Université Claude Bernard Lyon 1, Institut des Sciences Analytiques-UMR CNRS 5280, Villeurbanne, France; ^6^ Theoretical and Computational Chemistry Center, Institute of Applied Chemical Sciences, Faculty of Engineering, Universidad Autonoma de Chile, Santiago, Chile; ^7^ Departamento de Fisica, Facultad de Ciencias, Universidad de Chile, Santiago, Chile; ^8^ Centro para el desarrollo de la Nanociencias y Nanotecnologia, CEDENNA, Santiago, Chile

**Keywords:** chemical reactivity, density functional theory, molecular Interaction analysis, conceptual chemistry, response function

## Abstract

Reactivity descriptors indicate where a reagent is most reactive and how it is most likely to react. However, a reaction will only occur when the reagent encounters a suitable reaction partner. Determining whether a pair of reagents is well-matched requires developing reactivity rules that depend on both reagents. This can be achieved using the expression for the minimum-interaction-energy obtained from the density functional reactivity theory. Different terms in this expression will be dominant in different circumstances; depending on which terms control the reactivity, different reactivity indicators will be preferred.

## Graphical Abstract

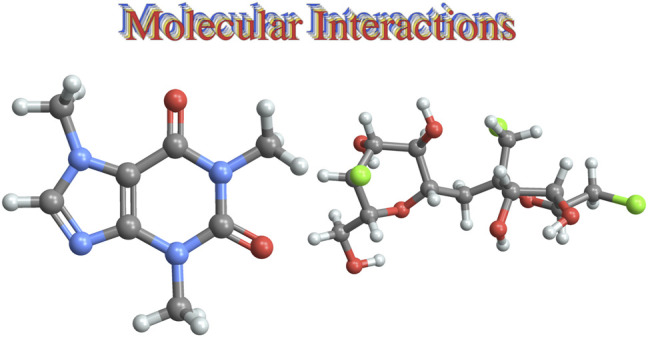

## 1 Introduction

When a reagent approaches a reactant molecule, the reactant is perturbed. If the perturbing reagent stabilizes the molecule (lowering its energy) or destabilizes the molecule comparatively little (raising its energy by a relatively small amount), then a reaction may occur. For example, regioselectivity can be predicted by finding the location where the reagent imparts the greatest additional stability to the reaction substrate. By comparing results for a series of reagents and a given molecule, or for a series of molecules and a single reagent, absolute chemical reactivity trends can be understood, and even predicted.

This basic viewpoint on chemical reactivity is the fundamental basis of the density functional theory for chemical reactivity (DFT-CR), often called chemical density functional theory or conceptual DFT (Parr and Yang, 1989; Chermette, 1999; Geerlings et al., 2003; Ayers et al., 2005a; Chattaraj et al., 2006; Gazquez, 2008; Liu, 2009a; Johnson et al., 2012b; De Proft et al., 2014; Fuentealba and Cardenas, 2015; Miranda-Quintana, 2018). The fundamental mathematics of DFT-CR is functional perturbation theory (Ayers et al., 2005a) using whichever representation of the chemical system (or “ensemble”) is deemed to be most convenient (Nalewajski and Parr, 1982; DeProft et al., 1997; Liu and Parr, 1997; Perez et al., 2008; Cardenas et al., 2009a). In this paper we will only consider the most common, “closed-system picture” (canonical ensemble). However, the same approach could be used for any of its Legendre (Johnson et al., 2012a) transforms (Ayers and Fuentealba, 2009).

In the closed-system picture, the state of a molecule is specified by its number of electrons, *N*, and the external potential that binds those electrons, *v*(**r**). When a reagent approaches, the number of electrons changes (from electron transfer between reagents) and the external potential changes (because the electrons in the molecule are attracted to the nuclei and repelled by the electrons of the approaching reagent). The change in the energy of a molecule with non-degenerate ground state (Cardenas et al., 2011a; Bultinck et al., 2013, 2014; Bultinck et al., 2015) is then,

$$\begin{array}{c} \Delta U\left[\Delta v\left(r\right);\Delta N\right]=\Delta V_{nn}+\left(\mu \Delta N+\frac{1}{2}\eta {\left(\Delta N\right)}^{2}+\cdots \right)\\ +\left(\Delta N\int f\left(r\right)\Delta v\left(r\right)dr+\frac{1}{2}{\left(\Delta N\right)}^{2}\int \Delta f\left(r\right)\Delta v\left(r\right)dr+\cdots \right)\\ \vdots \\ +\left(\int \rho \left(r\right)\Delta v\left(r\right)dr+\frac{1}{2}\iint \chi \left(r,r^{\prime }\right)\Delta v\left(r\right)\Delta v\left(r^{\prime }\right)drdr^{\prime }+\cdots \right) \end{array}$$ (1)

Here Δ*V*_*nn*_ is the change in nuclear-nuclear repulsion energy, Δ*N* is the number of electrons transferred from the reagent to the molecule under scrutiny and Δ*v*(**r**) is the change in the molecular external potential due to the presence of the reagent. These quantities depend on the particular identity of the reagent. The coefficients in this expansion—the chemical potential μ, (Parr et al., 1978) the chemical hardness η, (Parr and Pearson, 1983; Pearson, 1997; Ayers, 2007b) the Fukui function *f*(**r**), (Parr and Yang, 1984; Yang et al., 1984; Ayers and Levy, 2000; Ayers P. W. et al., 2009) the dual descriptor Δ*f*(**r**), (Fuentealba and Parr, 1991; Morell et al., 2005, 2006; Ayers et al., 2007; Cardenas et al., 2009b; Geerlings et al., 2012) the electron density ρ(**r**), and the linear-response (or polarizability) kernel χ(**r**,**r’**) (Berkowitz and Parr, 1988; Senet, 1996; Ayers, 2001; Sablon et al., 2010; Geerlings et al., 2014; Franco-Perez et al., 2015a)—are often taken to depend only on which molecule we are studying, and not on what reagent is being considered. The coefficients in the expansion determine how much (or how little) the reactant of interest is stabilized by a given reagent. Since energetically favorable interactions are associated with high reactivity, the coefficients in this expansion are reactivity indicators for the molecule being studied (Ayers et al., 2005a).

The assumption that the fine details of the reagent do not control the qualitative aspects of the reactivity is implicitly invoked when the coefficients of the Taylor expansion are used as reactivity indicators. This is often an adequate assumption. The mere idea that we can define scales of acidity and basicity (proclaiming, for example, that hydrochloric acid is a stronger acid than acetic acid) presumes that the properties of reactants (in this case, the acidity) do not depend very strongly on the identity of the attacking reagents (the base) or the solvent in which the reaction occurs. Similarly, when we say that nitrobenzene is a deactivating meta-director for electrophilic aromatic substitution we imply that the regioselectivity properties of nitrobenzene are largely independent of the choice of electrophile. Extending these ideas: most molecules are reactive at just one (and certainly no more than a few) molecular site(s); these reactivity preferences recur across a wide range of reagents, solvents, and experimental conditions. In cases like this, just a few coarse details of the reagent (e.g., the reagent’s electronegativity and hardness, which determine whether Δ*N* is small or large) and the reagent’s charge will suffice to describe the full range of possible reactions. In these cases, applying DFT-CR is as simple, and as complicated, as finding an appropriate coefficient (or linear combination of coefficients) from Eq. 1. The “perturbative perspective” on chemical reactivity says by choosing simple “model reagents” (often just a point charge with some capacity for accepting/donating electrons), one can decipher the reactivity preferences of the molecule of interest. There are many examples where this approach has been successfully employed to define new reactivity indicators (Parr et al., 1999; Ayers and Parr, 2000; Ayers and Parr, 2001; Ayers et al., 2005b; Anderson et al., 2007a; Anderson et al., 2007b; Cedillo et al., 2007; Gazquez et al., 2007; Cardenas et al., 2011b; Cardenas, 2011; Alain Miranda-Quintana R. and Ayers P. W., 2016), (Bochicchio, 2015; Alain Miranda-Quintana et al., 2016; Miranda-Quintana R. A., 2016, Miranda-Quintana, R. A. 2017; Gonzalez et al., 2018). This simple perspective has been successfully applied to the study of redox reactions, the study of solvation processes, and to the reactivity of aromatic species (Miranda-Quintana and Smiatek, 2020; Miranda-Quintana et al., 2022).

Chemistry is not always this simple. Certainly some reactions happen (or at least happen more readily than expected) because the reactant molecules are well-matched. Classic cases include the hard/soft acid/base principle and the Woodward-Hoffmann rules (e.g., ethene and butadiene are chemically similar, yet ethene is susceptible to cycloaddition with butadiene but not with itself). In the case of the hard/soft acid/base principle, treating both reagents as simplified “model reagents” suffices (Ayers, 2005; Ayers et al., 2006; Anderson et al., 2007a; Ayers, 2007b; Cardenas and Ayers, 2013; Miranda-Quintana, 2017a). The Woodward-Hoffmann rules can be treated using the DFT-CR framework, (De Proft et al., 2006; Ayers et al., 2007; De Proft et al., 2008; Sablon et al., 2009; Geerlings et al., 2012) but the fundamental *reason* that the dual descriptor and the initial hardness response suffice is still somewhat mysterious (Cardenas et al., 2009b). A more complete model for the interaction energy is clearly needed in this case. In the next section we will define just such a model. Section 3 discusses the key results. In follow-up papers we will derive an expression for the initial hardness response and reveal a new link between this quantity and the dual descriptor.

## 2 An Interaction Energy Model From Density Functional Theory Perturbation Theory

Our analysis is based on Eq. 1, including only the terms that are shown explicitly. Higher-order derivatives with respect to the number of electrons are usually small. (Fuentealba and Parr, 1991; Geerlings and De Proft, 2008; Morell et al., 2013; Heidar-Zadeh et al., 2016b; Munoz and Cardenas, 2017), and although they have attracted some recent interest, we will not take them into account here (Hoffmann et al., 2020; Miranda-Quintana R. A. et al., 2021; Miranda-Quintana R. A. et al., 2021). One may argue that since there are only one-body and two-body terms in the Hamiltonian, the energy and its functional derivatives with respect to *v*(**r**) should be nearly quadratic in the number of electrons (Ayers and Parr, 2008). Higher order derivatives with respect to the external potential correspond to hyperpolarizabilities; (Fuentealba and Parr, 1991; Senet, 1996; Cardenas et al., 2009a) these effects are difficult to compute accurately and are hopefully negligible. We assume that all the derivatives exist. Treating them exactly requires working with the system at finite temperature: (Franco-Perez et al., 2015a; Franco-Perez et al., 2015b; Malek and Balawender, 2015; Franco-Perez et al., 2016; Franco-Perez et al., 2017a; b;Franco-Perez et al., 2017c; Franco-Perez et al., 2017d; Franco-Perez et al., 2017e; Miranda-Quintana, 2017c; Miranda-Quintana RA. et al., 2017; Miranda-Quintana R. A. et al., 2017; Polanco-Ramirez et al., 2017; Franco-Pérez et al., 2018; Robles et al., 2018; Gázquez et al., 2019): since there are derivative discontinuities at the zero-temperature limit (Perdew et al., 1982; Yang et al., 2000; Ayers, 2008; Yang et al., 2016). One may argue that, for interacting molecular fragments, the “effective temperature” is nonzero; cf. refs. (Ayers, 2007a; Alain Miranda-Quintana R. and Ayers P. W., 2016).

The perturbation expansion, (1), gives us information about the energy of *A* in the presence of *B* $\left(U_{A}+\Delta U_{A}\left[\Delta v_{A}\left(r\right);\Delta N_{A}\right]\right)$ and the energy of *B* in the presence of *A* $\left(U_{B}+\Delta U_{B}\left[\Delta v_{B}\left(r\right);-\Delta N_{A}\right]\right)$ . We need to add these two interaction-energy expression and make corrections for 1) the double-counting of interactions and 2) nonadditive energy contributions like exchange and correlation between *A* and *B*.

The electron density of the interacting reagents, ρ_*AB*_(**r**), is divided into electron densities for the subsystems,

$$\rho_{AB}\left(r\right)=\rho_{A}\left(r\right)+\rho_{B}\left(r\right).$$ (2)

It is convenient to also define the nuclear charge density of the reactants, i.e.,

$$\begin{array}{l} z_{A}^{0}\left(r\right)=\sum_{\alpha \in A}Z_{\alpha }\delta \left(r-R_{\alpha }\right)\\ z_{B}^{0}\left(r\right)=\sum_{\beta \in B}Z_{\beta }\delta \left(r-R_{\beta }\right) \end{array}$$ (3)

The total charge density of reactant *A* is then $z_{A}^{0}\left(r\right)-\rho_{A}\left(r\right)$ . There is a similar expression for reactant *B*. The external potentials of the reactants are given by expressions like

$$v_{A}^{0}\left(r\right)=-\int \frac{z_{A}^{0}\left(r^{\prime }\right)}{\left|r-r^{\prime }\right|}dr^{\prime }$$ (4)

The interaction energy can then be written in terms of the fundamental density functional for the energy, (Hohenberg and Kohn, 1964)

$$\Delta U_{AB}=\Delta V_{nn}+\left(E_{v_{A}^{0}+v_{B}^{0}}\left[\rho_{AB}\right]-E_{v_{A}^{0}}\left[\rho_{A}^{0}\right]-E_{v_{B}^{0}}\left[\rho_{B}^{0}\right]\right)$$ (5)

Here $\rho_{A}^{0}\left(r\right)$ and $\rho_{B}^{0}\left(r\right)$ denote the electron densities of the separated reactants and

$$\Delta V_{nn}=\int \frac{z_{A}^{0}\left(r\right)z_{B}^{0}\left(r^{\prime }\right)}{\left|r-r^{\prime }\right|}drdr^{\prime }$$ (6)

denotes the change in nuclear-nuclear repulsion energy when the reactants are brought together. The contribution from rearrangements of the nuclei within the fragments is neglected for simplicity; this assumes that in the initial approach between the reagents, the internal molecular geometries of the individual reagents are preserved. This approximation was suggested, for example, in ref. (Ayers and Parr, 2001). This approximation can be relaxed if a term corresponding to the “preparation” energy of the reagents is added (Morokuma, 1971; Kitaura and Morokuma, 1976; Ziegler and Rauk, 1977, 1979; Wu et al., 2009; Azar and Head-Gordon, 2012).

The electronic energy functional is then decomposed,

$$\Delta U_{AB}=\Delta V_{nn}+\left(\begin{array}{l} T_{s}\left[\rho_{AB}\right]+E_{xc}\left[\rho_{AB}\right]+\int \rho_{AB}\left(r\right)\left(v_{A}^{0}\left(r\right)+v_{B}^{0}\left(r\right)\right)dr\\ +\frac{1}{2}\iint \frac{\rho_{AB}\left(r\right)\rho_{AB}\left(r^{\prime }\right)}{\left|r-r^{\prime }\right|}drdr^{\prime }-E_{v_{A}^{0}}\left[\rho_{A}^{0}\right]-E_{v_{B}^{0}}\left[\rho_{B}^{0}\right] \end{array}\right).$$ (7)

Here

$$F\left[\rho \right]=T_{s}\left[\rho \right]+J\left[\rho \right]+E_{xc}\left[\rho \right]$$ (8)

is the Hohenberg-Kohn functional, (Hohenberg and Kohn, 1964) $T_{s}\left[\rho \right]$ is the Kohn-Sham kinetic energy functional, (Kohn and Sham, 1965) and $E_{xc}\left[\rho \right]$ is the exchange-correlation energy functional.

$$E_{v}\left[\rho \right]=F\left[\rho \right]+\int \rho \left(r\right)v\left(r\right)dr$$ (9)

is the variational energy functional in DFT (Hohenberg and Kohn, 1964; Kohn and Sham, 1965)

Equation 7 can be simplified by breaking each contribution to the energy into its subsystem contributions:

$$\Delta U_{AB}=\Delta V_{nn}+\left(\begin{array}{l} T_{s}\left[\rho_{A}\right]+T_{s}\left[\rho_{B}\right]+\left\{T_{s}\left[\rho_{AB}\right]-T_{s}\left[\rho_{A}\right]-T_{s}\left[\rho_{B}\right]\right\}\\ +E_{xc}\left[\rho_{A}\right]+E_{xc}\left[\rho_{B}\right]+\left\{E_{xc}\left[\rho_{AB}\right]-E_{xc}\left[\rho_{A}\right]-E_{xc}\left[\rho_{B}\right]\right\}\\ \int \rho_{A}\left(r\right)v_{A}^{0}\left(r\right)dr+\int \rho_{B}\left(r\right)v_{B}^{0}\left(r\right)dr\\ +\int \left(\rho_{A}\left(r\right)v_{B}^{0}\left(r\right)+\rho_{B}\left(r\right)v_{A}^{0}\left(r\right)\right)dr\\ +\frac{1}{2}\iint \frac{\rho_{A}\left(r\right)\rho_{A}\left(r^{\prime }\right)}{\left|r-r^{\prime }\right|}drdr^{\prime }+\frac{1}{2}\iint \frac{\rho_{B}\left(r\right)\rho_{B}\left(r^{\prime }\right)}{\left|r-r^{\prime }\right|}drdr^{\prime }\\ +\iint \frac{\rho_{A}\left(r\right)\rho_{B}\left(r^{\prime }\right)}{\left|r-r^{\prime }\right|}drdr^{\prime }\\ -E_{v_{A}^{0}}\left[\rho_{A}^{0}\right]-E_{v_{B}^{0}}\left[\rho_{B}^{0}\right] \end{array}\right)$$ (10)

Using the definition of the electronic energy functional and Eq. 4, this simplifies to

$$\Delta U_{AB}=\left(\begin{array}{l} T_{s}\left[\rho_{AB}\right]-T_{s}\left[\rho_{A}\right]-T_{s}\left[\rho_{B}\right]\\ +E_{xc}\left[\rho_{AB}\right]-E_{xc}\left[\rho_{A}\right]-E_{xc}\left[\rho_{B}\right]\\ +\iint \frac{\left(z_{A}^{0}\left(r\right)-\rho_{A}\left(r\right)\right)\left(z_{B}^{0}\left(r^{\prime }\right)-\rho_{B}\left(r^{\prime }\right)\right)}{\left|r-r^{\prime }\right|}drdr^{\prime }\\ E_{v_{A}^{0}}\left[\rho_{A}\right]-E_{v_{A}^{0}}\left[\rho_{A}^{0}\right]+E_{v_{B}^{0}}\left[\rho_{B}\right]-E_{v_{B}^{0}}\left[\rho_{B}^{0}\right] \end{array}\right)$$ (11)

The terms in the first two lines are identified as the non-additive kinetic energy and the non-additive exchange-correlation energy,

$$T_{s}^{\text{non}-\text{add}}\left[\rho_{A},\rho_{B}\right]\equiv T_{s}\left[\rho_{AB}\right]-T_{s}\left[\rho_{A}\right]-T_{s}\left[\rho_{B}\right]$$ (12)

$$E_{xc}^{\text{non}-\text{add}}\left[\rho_{A},\rho_{B}\right]\equiv E_{xc}\left[\rho_{AB}\right]-E_{xc}\left[\rho_{A}\right]-E_{xc}\left[\rho_{B}\right]$$ (13)

The next term in Eq. 11 is the “non-additive Coulomb energy” this models the electrostatic interactions between the systems. The final terms in Eq. 11 include the polarization and electron-transfer energies. When the reactants approach each other, the electron densities of the reactants are polarized and the number of electrons in the reactants changes; the terms on the last line correct for these effects. In this approach, dispersion is treated as a facet of polarization.

The electrostatic interactions are readily evaluated in any quantum chemistry program, so the only “difficult” terms are the electron-transfer terms on the last line of Eq. 11. These could be approximated using a quadratic expansion in the electron density, i.e., (Liu and Parr, 1997; Ayers and Parr, 2000)

$$\begin{array}{c} E_{v_{A}^{0}}\left[\rho_{A}\right]-E_{v_{A}^{0}}\left[\rho_{A}^{0}\right]\approx \int \left(\rho_{A}\left(r\right)-\rho_{A}^{0}\left(r\right)\right)\mu_{A}^{0}dr\\ +\frac{1}{2}\iint \left(\rho_{A}\left(r\right)-\rho_{A}^{0}\left(r\right)\right)\eta \left[\rho_{A}^{0};r,r^{\prime }\right]\left(\rho_{A}\left(r^{\prime }\right)-\rho_{A}^{0}\left(r^{\prime }\right)\right)drdr^{\prime }\\ =\left(\Delta N_{A}\right)\mu_{A}^{0}\\ +\frac{1}{2}\iint \Delta \rho_{A}\left(r\right)\Delta \rho_{B}\left(r^{\prime }\right)\eta \left[\rho_{A}^{0};r,r^{\prime }\right]drdr^{\prime } \end{array}$$ (14)

The second-order derivative is the hardness kernel, (Berkowitz and Parr, 1988)

$$\eta \left[\rho ,r,r^{\prime }\right]=\frac{\delta F\left[\rho \right]}{\delta \rho \left(r\right)\delta \rho \left(r^{\prime }\right)}$$ (15)

The chemical potential and the hardness kernel of the isolated reagent could be evaluated exactly from the output of a Kohn-Sham program or using one of the many approximate forms that have been proposed in the literature (Liu et al., 1997; De Proft et al., 1998; Ayers, 2001; Chattaraj and Maiti, 2001; Torrent-Sucarrat et al., 2005; Torrent-Sucarrat et al., 2007; Gómez et al., 2021). Inserting (14) into (11) gives

$$\Delta U_{AB}\approx \left(\begin{array}{c} T_{s}^{\text{non}-\text{add}}\left[\rho_{A}^{0}+\Delta \rho_{A},\rho_{B}^{0}+\Delta \rho_{B}\right]+E_{xc}^{\text{non}-\text{add}}\left[\rho_{A}^{0}+\Delta \rho_{A},\rho_{B}^{0}+\Delta \rho_{B}\right]\\ +\Delta V_{nn}+\int \left(\Delta \rho_{A}\left(r\right)\Phi_{B}\left(r\right)+\Delta \rho_{B}\left(r\right)\Phi_{A}\left(r\right)\right)dr\\ -\iint \frac{\Delta \rho_{A}\left(r\right)\Delta \rho_{B}\left(r^{\prime }\right)}{\left|r-r^{\prime }\right|}drdr^{\prime }+\left(\mu_{A}^{0}-\mu_{B}^{0}\right)\Delta N_{A}\\ +\frac{1}{2}\iint \left[\begin{array}{l} \Delta \rho_{A}\left(r\right)\Delta \rho_{A}\left(r^{\prime }\right)\eta \left[\rho_{A}^{0};r,r^{\prime }\right]\\ +\Delta \rho_{B}\left(r\right)\Delta \rho_{B}\left(r^{\prime }\right)\eta \left[\rho_{B}^{0};r,r^{\prime }\right] \end{array}\right]drdr^{\prime } \end{array}\right)$$ (16)

In order to obtain a simple and intuitive form in Eq. 16, we have defined the electrostatic potential of the reactants in the usual way, e.g.,

$$\Phi_{A}\left(r\right)=\int \frac{z_{A}^{0}\left(r^{\prime }\right)-\rho_{A}^{0}\left(r^{\prime }\right)-\Delta \rho_{A}\left(r^{\prime }\right)}{\left|r-r^{\prime }\right|}dr^{\prime }.$$ (17)

with

$$\Delta \rho_{A}\left(r\right)=\rho_{A}\left(r\right)-\rho_{A}^{0}\left(r\right)$$ (18)

The actual interaction energy is then approximated by minimizing the expression in Eq. 16 with respect to all changes in density that preserve the total number of electrons and which do not cause the electron density of either fragment to become negative. i.e.,

$$\Delta U_{AB}\approx \underset{\Delta \rho_{A}\left(r\right);\Delta \rho_{B}\left(r\right)}{\underset{︸}{\min}}\Delta U_{AB}\left[\Delta \rho_{A},\Delta \rho_{B}\right]$$ (19)

subject to

$$\begin{array}{l} \rho_{A}^{0}\left(r\right)+\Delta \rho_{A}\left(r\right)\geq 0\\ \rho_{B}^{0}\left(r\right)+\Delta \rho_{B}\left(r\right)\geq 0 \end{array}$$ (20)

$$\Delta N_{A}\equiv \int \Delta \rho_{A}\left(r\right)dr=-\int \Delta \rho_{B}\left(r\right)dr$$ (21)

Equation 19 extends the variational principle for the Fukui function to include effects other than electron transfer (Chattaraj et al., 1995; Ayers and Parr, 2000).

For computational purposes, the preceding approach is probably the most useful. For conceptual purposes, however, an alternative perspective that makes references to the most commonly used reactivity indicators may be preferred. Notice that many of the commonly employed reactivity indicators appear in the expression for the change in electron density. e.g.,

$$\begin{array}{c} \Delta \rho_{A}\left(r\right)=\left(\Delta N_{A}f_{A}\left(r\right)+\frac{{\left(\Delta N_{A}\right)}^{2}}{2}\Delta f_{A}\left(r\right)+\cdots \right)\\ +\left(\int \chi_{A}\left(r,r^{\prime }\right)\Delta v_{A}\left(r^{\prime }\right)dr^{\prime }+\Delta N_{A}\int {\left(\frac{\partial \chi_{A}\left(r,r^{\prime }\right)}{\partial N_{A}}\right)}_{v\left(r\right)}\Delta v_{A}\left(r^{\prime }\right)dr^{\prime }+\cdots \right)\\ +\cdots \\ \approx \Delta N_{A}f_{A}\left(r\right)+\frac{{\left(\Delta N_{A}\right)}^{2}}{2}\Delta f_{A}\left(r\right)+\Delta \rho_{A}^{\left(\text{pol}\right)}\left[\Delta v_{A}\left(r\right);r\right] \end{array}$$ (22)

Note that we have defined the change in the electron density of *A* due to polarization as $\Delta \rho_{A}^{\left(\text{pol}\right)}\left[\Delta v_{A}\left(r\right);r\right]$ .

Equation 22 is directly useful only if we know $\Delta v_{A}\left(r\right)$ . However, we do not know how the external potential of *A* changes due to the presence of the other reagent. (There is an exact formulation to compute $\Delta v_{A}\left(r\right)$ , but it is computationally demanding (Ayers, 2000; Ayers and Parr, 2001; Kiewisch et al., 2008; Roncero et al., 2008; Fux et al., 2010)) Among the various approximations that have been proposed, the simplest is to identify $\Delta v_{A}\left(r\right)$ with the electrostatic potential of molecule *B* (Ayers and Parr, 2001). (This is certainly acceptable when the reactants are reasonably far apart, so that the electrons in reactant *A* and reactant *B* can be considered distinguishable particles.) This simple approximate form may also be rationalized from Eq. 16, where the electrostatic potential of *B* serves as an “effective external potential” for the electrons in *A*.

Using

$$\begin{array}{l} \Delta v_{A}\left(r\right)=-\Phi_{B}\left(r\right)\\ \Delta v_{B}\left(r\right)=-\Phi_{A}\left(r\right) \end{array}$$ (23)

and Eq. 22, we can expand the electrostatic terms in Eq. 11, giving,

$$\begin{array}{l} \iint \frac{\left(z_{A}^{0}\left(r\right)-\rho_{A}\left(r\right)\right)\left(z_{B}^{0}\left(r\right)-\rho_{B}\left(r^{\prime }\right)\right)}{\left|r-r^{\prime }\right|}drdr^{\prime }\\ \approx \iint \frac{\left(z_{A}^{0}\left(r\right)-\rho_{A}^{0}\left(r\right)\right)\left(z_{B}^{0}\left(r^{\prime }\right)-\rho_{B}^{0}\left(r^{\prime }\right)\right)}{\left|r-r^{\prime }\right|}drdr^{\prime }\\ -\Delta N_{A}\int \left(f_{A}\left(r\right)\Phi_{B}^{0}\left(r\right)+f_{B}\left(r\right)\Phi_{A}^{0}\left(r\right)\right)dr\\ -\frac{{\left(\Delta N_{A}\right)}^{2}}{2}\int \left(\Delta f_{A}\left(r\right)\Phi_{B}^{0}\left(r\right)+\Delta f_{B}\left(r\right)\Phi_{A}^{0}\left(r\right)\right)dr\\ -\int \left(\Delta \rho_{A}^{\left(\text{pol}\right)}\left[\Phi_{B};r\right]\Phi_{B}^{0}\left(r\right)+\Delta \rho_{B}^{\left(\text{pol}\right)}\left[\Phi_{A};r\right]\Phi_{A}^{0}\left(r\right)\right)\\ +\iint \frac{\Delta \rho_{A}^{\left(\text{pol}\right)}\left[\Phi_{B};r\right]\Delta \rho_{B}^{\left(\text{pol}\right)}\left[\Phi_{A};r^{\prime }\right]}{\left|r-r^{\prime }\right|}drdr^{\prime }\\ -\Delta N_{A}\iint \frac{\Delta \rho_{A}^{\left(\text{pol}\right)}\left[\Phi_{B};r\right]f_{B}\left(r^{\prime }\right)-\Delta \rho_{B}^{\left(\text{pol}\right)}\left[\Phi_{A};r\right]f_{A}\left(r^{\prime }\right)}{\left|r-r^{\prime }\right|}drdr^{\prime }\\ +\frac{{\left(\Delta N_{A}\right)}^{2}}{2}\iint \frac{\Delta \rho_{A}^{\left(\text{pol}\right)}\left[\Phi_{B};r\right]\Delta f_{B}\left(r^{\prime }\right)+\Delta \rho_{B}^{\left(\text{pol}\right)}\left[\Phi_{A};r\right]\Delta f_{A}\left(r^{\prime }\right)}{\left|r-r^{\prime }\right|}drdr^{\prime }\\ -{\left(\Delta N_{A}\right)}^{2}\iint \frac{f_{A}\left(r\right)f_{B}\left(r^{\prime }\right)}{\left|r-r^{\prime }\right|}drdr^{\prime }\\ +{\left(\Delta N_{A}\right)}^{3}\iint \frac{f_{A}\left(r\right)\Delta f_{B}\left(r^{\prime }\right)-f_{B}\left(r\right)\Delta f_{A}\left(r^{\prime }\right)}{\left|r-r^{\prime }\right|}drdr^{\prime }\\ +\frac{{\left(\Delta N_{A}\right)}^{4}}{4}\iint \frac{\Delta f_{A}\left(r\right)\Delta f_{B}\left(r^{\prime }\right)}{\left|r-r^{\prime }\right|}drdr^{\prime } \end{array}$$ (24)

In addition to the contributions to the interaction energy from electrostatic interactions of the reactants and the electron transfer between them, there are contributions due to mutual polarization of the reactants. The leading order term of this type is

$$J_{\Delta \rho^{\left(\text{pol}\right)}}=-\int \left(\Delta \rho_{A}^{\left(\text{pol}\right)}\left[\Phi_{B};\Delta N_{A},r\right]\Phi_{B}^{0}\left(r\right)+\Delta \rho_{B}^{\left(\text{pol}\right)}\left[\Phi_{A};-\Delta N_{A},r\right]\Phi_{A}^{0}\left(r\right)\right)$$ (25)

This term is negative: the electrostatic potential of reactant *B* polarizes the electron density of *A* in a way that increases the attraction between the reagents.

Some readers may question whether $J_{\Delta \rho^{\left(\text{pol}\right)}}$ should be multiplied by a factor of 1/2. After all, the change in energy due to polarization is

$$\begin{array}{c} \Delta E_{\text{pol}}\approx \frac{1}{2}\iint \Delta v\left(r\right)\Delta v\left(r^{\prime }\right)\chi \left(r,r^{\prime }\right)drdr^{\prime }\\ \approx \frac{1}{2}\int \Delta \rho^{\left(\text{pol}\right)}\left[\Delta v;r\right]\Delta v\left(r\right)dr. \end{array}$$ (26)

When the two reactants approach each other, they polarize each other. This polarization increases the attraction of the reactants for each other. However, polarization also mixes excited-state wavefunctions with the ground-state wavefunction; this increases the electronic energy of the reactants(Ayers, 2007a). The total change in energy, cf. Eq. 11, arises because the electronic energy of the reactants, $E_{v_{A}^{0}}\left[\rho_{A}\right]$ and $E_{v_{B}^{0}}\left[\rho_{B}\right]$ , increases by $-\Delta E_{\text{pol}}$ while the interaction energy between the reactants decreases by twice that amount. This explains why there is no factor of 1/2 in Eq. 24 and draws our attention to the final contributions to the fundamental expression for the interaction energy, Eq. 11. When the electron density of the reactants changes (due to polarization or electron transfer), the energy of the reactants also changes. One has

$$\begin{array}{c} E_{v_{A}^{0}}\left[\rho_{A}\right]-E_{v_{A}^{0}}\left[\rho_{A}^{0}\right]=\left(\Delta N_{A}\mu_{A}+\frac{{\left(\Delta N_{A}\right)}^{2}}{2}\eta_{A}+\cdots \right)\\ +\frac{1}{2}\int \Delta \rho_{A}\left[\Phi_{B};\Delta N_{A},r\right]\Phi_{B}\left(r\right)dr+\cdots \end{array}$$ (27)

The terms in the first line represent the change in system energy due to electron transfer; the terms in the second line shows how the energy of the system increases as the density is polarized away from the ground-state density.

Combining Eqs 11, 22, 24, 27 gives an alternative method for computing the interaction energy between reagents.

**A.** Determine the non-additive kinetic energy and the non-additive exchange-correlation energy. Sometimes simple Thomas-Fermi-Dirac-like functionals will suffice here. One has

$$T_{s}^{\text{non}-\text{add}}\left[\rho_{A}^{0},\rho_{B}^{0}\right]\equiv T_{s}\left[\rho_{A}^{0}+\rho_{B}^{0}\right]-T_{s}\left[\rho_{A}^{0}\right]-T_{s}\left[\rho_{B}^{0}\right]$$ (28)

$$E_{xc}^{\text{non}-\text{add}}\left[\rho_{A}^{0},\rho_{B}^{0}\right]\equiv E_{xc}\left[\rho_{A}^{0}+\rho_{B}^{0}\right]-E_{xc}\left[\rho_{A}^{0}\right]-E_{xc}\left[\rho_{B}^{0}\right].$$ (29)

**B.** Compute the electrostatic potential of the isolated reactants. E.g.,

$$\Phi_{A}^{0}\left(r\right)=\int \frac{z_{A}^{0}\left(r^{\prime }\right)-\rho_{A}^{0}\left(r^{\prime }\right)}{\left|r-r^{\prime }\right|}dr^{\prime }.$$ (30)

Use the electrostatic potential to estimate how the density of each reactant is polarized by the electrostatic potential of the other reactant,

$$\Delta \rho_{A}^{\left(\text{pol}\right)}\left[\Phi_{B}^{0};r\right]=\int {\left(\frac{\delta \rho_{A}\left(r\right)}{\delta v\left(r^{\prime }\right)}\right)}_{N_{A}^{0}}\Phi_{B}^{0}\left(r^{\prime }\right)dr^{\prime }.$$ (31)

Also compute the polarization energy,

$$E_{A}^{\left(\text{pol}\right)}\left[\Phi_{B}^{0}\right]=-\frac{1}{2}\int \Delta \rho_{A}^{\left(\text{pol}\right)}\left[\Phi_{B}^{0};r\right]\Phi_{B}^{0}\left(r\right)dr.$$ (32)

**C.** Determine the extent of electron transfer, $\Delta N_{A}$ , by minimizing the expression for the interaction energy with respect to $\Delta N_{A}$ .

$$\begin{array}{c} \Delta U_{AB}\left[\Delta N_{A}\right]=\left(T_{s}^{\text{non}-\text{add}}\left[\rho_{A}^{0},\rho_{B}^{0}\right]+E_{xc}^{\text{non}-\text{add}}\left[\rho_{A}^{0},\rho_{B}^{0}\right]\right)\\ +\left(\begin{array}{c} \iint \frac{\left(z_{A}^{0}\left(r\right)-\rho_{A}^{0}\left(r\right)\right)\left(z_{B}^{0}\left(r^{\prime }\right)-\rho_{B}^{0}\left(r^{\prime }\right)\right)}{\left|r-r^{\prime }\right|}drdr^{\prime }\\ +2\left(E_{A}^{\left(\text{pol}\right)}\left[\Phi_{B}^{0}\right]+E_{B}^{\left(\text{pol}\right)}\left[\Phi_{A}^{0}\right]\right)\\ +\iint \frac{\Delta \rho_{A}^{\left(\text{pol}\right)}\left[\Phi_{B}^{0},r\right]\Delta \rho_{B}^{\left(\text{pol}\right)}\left[\Phi_{A}^{0},r^{\prime }\right]}{\left|r-r^{\prime }\right|}drdr^{\prime }\\ -\Delta N_{A}\int \left(f_{A}\left(r\right)\Phi_{B}^{0}\left(r\right)-f_{B}\left(r\right)\Phi_{A}^{0}\left(r\right)\right)dr\\ +\Delta N_{A}\iint \frac{\left(\begin{array}{l} \Delta \rho_{B}^{\left(\text{pol}\right)}\left[\Phi_{A}^{0},r\right]f_{A}\left(r^{\prime }\right)\\ -\Delta \rho_{A}^{\left(\text{pol}\right)}\left[\Phi_{B}^{0},r\right]f_{B}\left(r^{\prime }\right) \end{array}\right)}{\left|r-r^{\prime }\right|}drdr^{\prime }\\ -\frac{{\left(\Delta N_{A}\right)}^{2}}{2}\int \left(\Delta f_{A}\left(r\right)\Phi_{B}^{0}\left(r\right)+\Delta f_{B}\left(r\right)\Phi_{A}^{0}\left(r\right)\right)dr\\ +\frac{{\left(\Delta N_{A}\right)}^{2}}{2}\iint \frac{\left(\begin{array}{l} \Delta \rho_{A}^{\left(\text{pol}\right)}\left[\Phi_{B}^{0},r\right]\Delta f_{B}\left(r^{\prime }\right)\\ +\Delta \rho_{B}^{\left(\text{pol}\right)}\left[\Phi_{A}^{0},r\right]\Delta f_{A}\left(r^{\prime }\right) \end{array}\right)}{\left|r-r^{\prime }\right|}drdr^{\prime }\\ -{\left(\Delta N_{A}\right)}^{2}\iint \frac{f_{A}\left(r\right)f_{B}\left(r^{\prime }\right)}{\left|r-r^{\prime }\right|}drdr^{\prime }\\ +{\left(\Delta N_{A}\right)}^{3}\iint \frac{f_{A}\left(r\right)\Delta f_{B}\left(r^{\prime }\right)-f_{B}\left(r\right)\Delta f_{A}\left(r^{\prime }\right)}{\left|r-r^{\prime }\right|}drdr^{\prime }\\ +\frac{{\left(\Delta N_{A}\right)}^{4}}{4}\iint \frac{\Delta f_{A}\left(r\right)\Delta f_{B}\left(r^{\prime }\right)}{\left|r-r^{\prime }\right|}drdr^{\prime } \end{array}\right)\\ +\left(\begin{array}{l} \Delta N_{A}\left(\mu_{A}^{0}-\mu_{B}^{0}\right)+\frac{{\left(\Delta N_{A}\right)}^{2}}{2}\left(\eta_{A}^{0}+\eta_{B}^{0}\right)\\ -E_{A}^{\left(\text{pol}\right)}\left[\Phi_{B}^{0}\right]-E_{B}^{\left(\text{pol}\right)}\left[\Phi_{A}^{0}\right] \end{array}\right) \end{array}$$ (33)

**D.** The electron density of the reagents is now changed due to electron transfer and mutual polarization. Use the updated electron densities,

$$\rho_{A}^{1}\left(r\right)=\rho_{A}^{0}\left(r\right)+\Delta N_{A}^{0}f_{A}^{0}\left(r\right)+\frac{{\left(\Delta N_{A}^{0}\right)}^{2}}{2}\Delta f_{A}^{0}\left(r\right)+\Delta \rho_{A}^{\left(\text{pol}\right)}\left[\Phi_{B}^{0};r\right]$$ (34)

to compute an updated electrostatic potential $\Phi_{A}^{1}\left(r\right)$ . This can then be used to update the density change due to polarization and the polarization energy (using Eqs 31, 32; step B). The extent of electron transfer is now computed by substituting the updated quantities into (Eq. 33) and minimizing the energy with respect to $\Delta N_{A}$ (step C). The electron density of the reactants can then be updated

$$\rho_{A}^{2}\left(r\right)=\rho_{A}^{1}\left(r\right)+\Delta N_{A}^{1}f_{A}^{0}\left(r\right)+\frac{{\left(\Delta N_{A}^{1}\right)}^{2}}{2}\Delta f_{A}^{0}\left(r\right)+\Delta \rho_{A}^{\left(\text{pol}\right)}\left[\Phi_{B}^{1};r\right]$$ (35)

and steps B and C can be iterated until value of the interaction energy has converged to the desired level of precision. (Notice how the supra-indices in Eqs 34, 35 distinguish between the magnitudes that are updated, from those that are kept constant.)

Step A could also be included in the iteration cycle, but if that is done then one needs to use the linear response kernel for the perturbed molecular fragment when evaluating Eq. 31; this may not be practical.

In this form, we are always expanding the Taylor series given in Eq. 33 around a fixed point that corresponds to the isolated reagents. We can improve this procedure by centering the expansion at a point that already contains the initial interaction between the molecules or, even more generally, the influence of the molecular environement (e.g., solvent) (Alain Miranda-Quintana R. and Ayers P. W., 2016, Alain Miranda-Quintana R. and Ayers, P. W. 2016; Alain Miranda-Quintana et al., 2016). For example, we can correct the energies using:

$$E_{M}=E_{M}^{0}+\int \rho_{M}^{0}\left(r\right)\Delta v\left(r\right)dr$$ (36)

where the sub-index *M* indicates the number of electrons, and the supra-index 0 identifies the isolated-system properties. We can also update the density by considering the effect of the perturbation on the Kohn-Sham orbitals:

$$|\varphi_{k}\rangle =\sum_{p\neq k}\frac{\langle \varphi_{p}^{0}|\Delta v\left(r\right)|\varphi_{k}^{0}\rangle }{\varepsilon_{k}-\varepsilon_{p}}|\varphi_{p}^{0}\rangle$$ (37)

In these cases we take the perturbation, Δv(r) , according to Eqs 23, 30. The modified energies and densities can then be used to estimate the reactivity descriptors present in Eq. 33.

An even more realistic solution will be to change the point around which we will expand the Taylor expansion after each iteration step. To do so, we will have to recalculate the perturbed energies and orbitals (e.g., densities) after each iteration, using the electrostatic potentials associated to Eq. 35. But, additionally, we must correct the expressions used to calculate the chemical potentials and Fukui functions to accomodate fractionally-charged systems:

$$\mu_{M+\Delta N}=\mu_{M}+\eta_{M}\Delta N$$ (38)

$$f{\left(r\right)}_{M+\Delta N}=f{\left(r\right)}_{M}+\Delta f{\left(r\right)}_{M}\Delta N$$ (39)

Extending the preceding mathematical framework to include reagents with degenerate ground states is somewhat difficult, but is not impossible (Cardenas et al., 2011a; Bultinck et al., 2013, 2014; Bultinck et al., 2015). Extending this procedure to include open-shell reagents and spin-specific reaction pathways is quite straightforward: (Galvan et al., 1988; Galvan and Vargas, 1992; Ghanty and Ghosh, 1994; Ghosh, 1994; Ghanty and Ghosh, 1996; Vargas and Galvan, 1996; Vargas et al., 1998; Melin et al., 2003; Miranda-Quintana R. A. and Ayers P. W., 2016): none of the formulas change if the matrix-vector notation for spin-resolved reactivity indicator functions is invoked (Garza et al., 2006; Perez et al., 2008).

## 3 Discussion

### 3.1 Connections to Other Theoretical Methods

This method of computing the interaction energy from its fragment decomposition is fundamentally related to the method of density-functional embedding (Cortona, 1991; Vaidehi et al., 1992; Wesolowski and Warshel, 1993; Govind et al., 1999; Wesolowski, 2004; Wesolowski and Leszczynski, 2006; Elliott et al., 2010; Fux et al., 2010; Goodpaster et al., 2010; Goodpaster et al., 2011; Laricchia et al., 2011). That method, unlike this one, performs iterative *ab initio* calculations on the fragments rather than using the perturbation expansion.

This method is also fundamentally related to electronegativity equalization approaches to molecular mechanics (Mortier et al., 1985; Mortier et al., 1986; Yang and Mortier, 1986; Mortier, 1987; Rappe and Goddard, 1991; Bultinck et al., 2002a; Bultinck et al., 2002b; Verstraelen et al., 2009; Verstraelen et al., 2011). In those methods one replaces the non-additive kinetic and exchange-correlation energies with an empirically parameterized “repulsive wall.” One accelerates the method by approximating the Coulomb-like integrals by using atom-centered charges and (sometimes) dipoles, with the charges and dipoles varying in response to external fields (polarization) and electron transfer to/from the molecule; this amounts to using a coarse-grained version of Eq. 16. The result is a relatively fast and accurate method for computing molecular interaction energies. In this latter context it bears mention that both of the fundamental expressions for the interaction energy, Eqs 16, 33, can be adapted to cases where more than two molecules need to be considered (e.g., two reactants and a catalyst, several reactant molecules and the surrounding solvent). Assuming that three-body forces are negligible, the interaction energy in these more complicated situations is merely the sum over all the pairwise interaction energies.

This approach is fundamentally related to the electron-density-based energy decomposition analysis (DFT-EDA) (Wu et al., 2009). In DFT-EDA, two fragments are brought together with frozen density (corresponding to the first line in Eqs 16, 33) and then the *exact* (nonperturbative) electron-transfer energy and polarization-energies are defined and computed using constrained search computations. Like the approach in this paper, DFT-EDA is intended to elucidate chemistry, not to provide numbers. This approach, however, is mainly intended to *predict* chemistry in the perturbative regime, while DFT-EDA is generally employed for *understanding* known chemistry using an exact (nonperturbative) expression for the energy.

Finally, this approach is partly justified by the nearsightedness principle in density-functional theory (Li et al., 1993; Kohn, 1996; Baer and Head-Gordon, 1997; Prodan and Kohn, 2005; Prodan, 2006). It is safe to assume that when two reactants are far from each other, the “long-range” effects are mainly electron transfer and electrostatics, and that large-scale induced electron rearrangements are minimal (Fias et al., 2017). This means, in particular, that the estimate of the polarization density in step B, which implicitly assumes that the response of one subsystem to the other is small, and that the (total system) linear response kernel, $\chi_{AB}\left(r,r^{\prime }\right)$ , and higher-order responses is negligible (except perhaps for features described by electron transfer and the Fukui function/dual descriptor) when r and $r^{\prime }$ are located on different subsystems (Cardenas et al., 2009b; Fias et al., 2017). That is, in step (B) we assume that the response kernel is nearsighted enough for the approximation $\chi_{AB}\left(r,r^{\prime }\right)\approx \chi_{A}\left(r,r^{\prime }\right)+\chi_{B}\left(r,r^{\prime }\right)$ to be valid. A similar nearsightedness argument applies to the hardness kernel in the alternative expression for the interaction energy in Eq. 16.

### 3.2 Connections to Previous Results in Density Functional Theory for Chemical Reactivity

The two expressions for the interaction energy, Eqs 16, 33, are the key result of this paper. In certain limits, these expressions recover known results from the DFT-CR literature. For example, suppose that the reactivity trends in fragment *A* do not depend on the detailed properties of fragment *B*. Then we can neglect the terms in Eq. 16 that depend on fragment *B*, obtaining,

$$\Delta U_{AB}\approx \frac{1}{2}\iint \Delta \rho_{A}\left(r\right)\Delta \rho_{A}\left(r^{\prime }\right)\eta \left[\rho_{A}^{0};r,r^{\prime }\right]drdr^{\prime }$$ (40)

Minimizing this expression with respect to all $\Delta \rho_{A}\left(r\right)$ with Δ*N*_*A*_ electrons is equivalent to the variational principle for the Fukui function (Chattaraj et al., 1995; Ayers and Parr, 2000). This establishes the Fukui function as the key nonspecific reactivity indicator for electron-transfer reactivity. Recall that the lowest-curvature normal modes on a potential energy surface correspond to the most facile molecular rearrangements. From Eq. 40 we can approximate the elements of the Hessian matrix as,

$$\frac{\partial U_{AB}}{\partial R_{i}\partial R_{j}}=\frac{1}{2}\iint \frac{\partial \rho_{A}\left(r\right)}{\partial R_{i}}\frac{\partial \rho_{A}\left(r^{\prime }\right)}{\partial R_{j}}\eta \left[\rho_{A}^{0};r,r^{\prime }\right]drdr^{\prime }.$$ (41)

This suggests that eigenvectors of the hardness kernel with small eigenvalues indicate favorable reactive modes. Or, to phrase this in the more conventional way: the eigenvectors of the softness kernel with the highest eigenvalues indicate favorable molecular rearrangements (Nalewajski and Koninski, 1987; Cohen et al., 1995; Cardenas et al., 2006). These simple guiding principles based on Eq. 16 obviously fail when semiquantitative results are needed or when detailed “matching” of reactants plays a pivotal role in the chemical reaction.

Equation 33 may seem very intimidating, but its interpretation is relatively simple. There are strong links to the previous two-reagent expressions in the literature, especially from the work of Parr and Pearson (for electron transfer), (Parr and Pearson, 1983) Berkowitz (for Frontier-orbital interactions), (Berkowitz, 1987) Anderson *et al.* (for the balance between electrostatics and electron-transfer terms), (Anderson et al., 2007a) and Morell *et al.* (for electrophile/nucleophile matching) (Morell et al., 2005; Ayers et al., 2007; Morell et al., 2007; Morell et al., 2008; Cardenas et al., 2009b; Labet et al., 2009). For example, if one assumes that all regioselective interactions may be neglected, then the entire expression (33) reduces to the conventional Parr-Pearson quadratic expansion for the electron-transfer energy, (Parr and Pearson, 1983; Ayers et al., 2006)

$$\Delta U_{AB}\left[\Delta N_{A}\right]\approx \Delta N_{A}\left(\mu_{A}^{0}-\mu_{B}^{0}\right)+\frac{{\left(\Delta N_{A}\right)}^{2}}{2}\left(\eta_{A}^{0}+\eta_{B}^{0}\right).$$ (42)

If one furthermore chooses one of the reagents to be a perfect electron donor, the change in energy is equal to the electrophilicity (Parr et al., 1999; Chattaraj et al., 2003; Chattaraj et al., 2006; Liu, 2009b).

If electron transfer is dominant and one wishes to consider regioselectivity, then the leading-order term is the Coulomb attraction between the Fukui functions,

$$J_{f}\equiv -{\left(\Delta N_{A}\right)}^{2}\iint \frac{f_{A}\left(r\right)f_{B}\left(r^{\prime }\right)}{\left|r-r^{\prime }\right|}drdr^{\prime }$$ (43)

This term plays a decisive role in the theory of Frontier-molecular orbital interactions put forth by Berkowitz (Berkowitz, 1987) and it justifies the use of the Fukui potential as a fundamental reactivity indicator (Cardenas et al., 2011b; Cardenas, 2011; Osorio et al., 2011; Yañez et al., 2021). When only the potential is varied, but not the number of electrons, one obtains potential-based reactivity indicators (Ayers and Parr, 2001; Cedillo et al., 2007; Ayers P. W. et al., 2009; Liu et al., 2009; Muñoz et al., 2020).

### 3.3 Term-By-Term Interpretation of Eq. 33

The Taylor series expansion in Eq. 1 converges subject to reasonable assumptions (Ayers et al., 2005a). Equation 33 is therefore approximate only in two ways: 1) higher order terms in the Taylor series are neglected and 2) only electrostatic effects are included in the model for the external potential change. Both assumptions are accurate for distant, weakly-interacting, reagents with nondegenerate ground states; they will be useful in all cases where the initial approach of the reagents guides the reactivity preferences (e.g., when the transition state is early). Both assumptions can be relaxed. Many higher-order reactivity indicators are known, (Fuentealba and Parr, 1991; Senet, 1996; Geerlings and De Proft, 2008; Cardenas et al., 2009a; Heidar-Zadeh et al., 2016b) though they are difficult to compute and inconvenient to work with. The exact “effective external potential” that a molecule feels in the presence of a reagent can be computed, and various levels of approximation are known (Ayers and Parr, 2001). Some of these approximations are not that much more complicated than the simple electrostatic approximation. (For example, including an “exchange-correlation charge density” is relatively straightforward (Liu et al., 1999; Ayers and Levy, 2001; Ayers and Parr, 2001; Andrade and Aspuru-Guzik, 2011)) Even with the approximations implicit in Eq. 33, however, the main chemical effects that drive reactions are included.

#### 3.3.1 Steric Repulsion and Electron Pairing

The non-additive kinetic and exchange-correlation energy terms,

$$T_{s}^{\text{non}-\text{add}}\left[\rho_{A}^{0},\rho_{B}^{0}\right]+E_{xc}^{\text{non}-\text{add}}\left[\rho_{A}^{0},\rho_{B}^{0}\right]$$ (44)

capture the effects of electon pairing (from combining the electron densities of the fragments) and, when closed shells are pushed together, capture the “Pauli” and “kinetic” portions of the repulsive wall in potential energy curves. The electrostatic repulsion between the fragments also contributes to steric hindrance; this contribution is included in the second line.

We note that this measure of the steric repulsion is different, both physically and mathematically, from the quantification of the steric effect proposed by one of us, (Liu, 2007) and then studied and extended by us and others (Liu, 2007; Nagy, 2007; Liu and Govind, 2008; Torrent-Sucarrat et al., 2009; Ugur et al., 2009; Ess et al., 2010; Liu et al., 2010; Tsirelson et al., 2010; Esquivel et al., 2011; Huang et al., 2011; Fang et al., 2014). Nonetheless, the two measures have some mathematical similarities (in both cases the dominant term is associated with the kinetic energy) and are expected to give similar insights. Note also that Eq. 44 also includes some long-range contributions associated with exchange and correlation and short-ranged contributions from electron pairing that are not true “steric” effects.

#### 3.3.2 Electrostatic Interactions

The electrostatic attractions/repulsions between the isolated fragments are accounted for in the term

$$\iint \frac{\left(z_{A}^{0}\left(r\right)-\rho_{A}^{0}\left(r\right)\right)\left(z_{B}^{0}\left(r^{\prime }\right)-\rho_{B}^{0}\left(r^{\prime }\right)\right)}{\left|r-r^{\prime }\right|}drdr^{\prime }.$$ (45)

Notice that, Eqs 44, 45 together can already describe weak interactions; they are the key elements in the Gordon-Kim theory, for example (Gordon and Kim, 1972).

#### 3.3.3 Polarization and Dispersion

The term

$$2\left(E_{A}^{\left(\text{pol}\right)}\left[\Phi_{B}^{0}\right]+E_{B}^{\left(\text{pol}\right)}\left[\Phi_{A}^{0}\right]\right)$$ (46)

accounts for the electrostatic stabilization that occurs because the fragments are polarized. (The very last line in Eq. 33 corrects for the “promotion energy” that is required to deform the electron density to make these favorable electrostatic interactions.) The second-order change in electrostatics due to the mutual polarization of the fragments is captured in the term

$$\iint \frac{\Delta \rho_{A}^{\left(\text{pol}\right)}\left[\Phi_{B}^{0},r\right]\Delta \rho_{B}^{\left(\text{pol}\right)}\left[\Phi_{A}^{0},r^{\prime }\right]}{\left|r-r^{\prime }\right|}drdr^{\prime }$$ (47)

If the interaction-energy expression is iteratively converged (step D in Section 2), this term includes the “induced multipole-induced multipole” interactions that are implicated in dispersion.

#### 3.3.4 Electron Transfer; Fukui Function

The Coulomb interaction between the Fukui functions (Parr and Yang, 1984; Yang et al., 1984; Ayers and Levy, 2000; Ayers P. W. et al., 2009; Osorio et al., 2011; Yañez et al., 2021) is the leading-order purely electron-transfer term,

$$-{\left(\Delta N_{A}\right)}^{2}\iint \frac{f_{A}\left(r\right)f_{B}\left(r^{\prime }\right)}{\left|r-r^{\prime }\right|}drdr^{\prime }$$ (48)

The importance of this term had already been noted by Berkowitz (Berkowitz, 1987). Electron transfer will be favorable when the Coulomb repulsion between the donor Fukui function and the acceptor Fukui function is large; this is associated with strong overlap of the Frontier orbitals. Alternatively, Eq. 48 can be viewed as the attraction between the donor orbital and the acceptor orbital, or between the electron and the hole (Ayers and Levy, 2000). Similar terms are found to play a role in Frontier orbital theories, especially when expansions around the separated atom limit are considered (De Proft et al., 2006; Cardenas et al., 2009b).

Electron transfer also changes the electrostatic interactions between reagents at both the isolated reagent,

$$-\Delta N_{A}\int \left(f_{A}\left(r\right)\Phi_{B}^{0}\left(r\right)-f_{B}\left(r\right)\Phi_{A}^{0}\left(r\right)\right)dr$$ (49)

and polarized reagent

$$+\frac{{\left(\Delta N_{A}\right)}^{2}}{2}\iint \frac{\left(\Delta \rho_{A}^{\left(\text{pol}\right)}\left[\Phi_{B}^{0},r\right]f_{B}\left(r^{\prime }\right)+\Delta \rho_{B}^{\left(\text{pol}\right)}\left[\Phi_{A}^{0},r\right]f_{A}\left(r^{\prime }\right)\right)}{\left|r-r^{\prime }\right|}drdr^{\prime }$$ (50)

levels. Terms similar to Eqs 48, 49 can be important even when electron transfer is small because the asymptotic behavior of the linear response function is governed by the Fukui function (Cardenas et al., 2009b; Fias et al., 2017).

#### 3.3.5 Electron Transfer; Dual Descriptor

The dual descriptor (Morell et al., 2005, 2006; Ayers et al., 2007; Morell et al., 2007; Geerlings et al., 2012) tends to play a role when the reagents are electrostatically flat and the Fukui functions do not establish a strong reactivity preference. The leading-order purely dual-descriptor term is

$$\frac{{\left(\Delta N_{A}\right)}^{4}}{4}\iint \frac{\Delta f_{A}\left(r\right)\Delta f_{B}\left(r^{\prime }\right)}{\left|r-r^{\prime }\right|}drdr^{\prime }.$$ (51)

This expression has appeared previously in the literature, but without derivation (Ayers et al., 2007). Notice that, unlike the interaction between the Fukui functions (Eq. 48), it is favorable to have a large electrostatic attraction between the dual descriptors of the fragments; this corresponds to aligning the nucleophilic portions of one fragment with the electrophilic portions of a different fragment, and vice versa (Morell et al., 2005, 2006; Ayers et al., 2007; Morell et al., 2007; Geerlings et al., 2012).

The change in electron density due to second-order electron transfer also causes a shift in the electrostatic interactions with the isolated (compare Eq. 49)

$$-\frac{{\left(\Delta N_{A}\right)}^{2}}{2}\int \left(\Delta f_{A}\left(r\right)\Phi_{B}^{0}\left(r\right)+\Delta f_{B}\left(r\right)\Phi_{A}^{0}\left(r\right)\right)dr$$ (52)

and polarized (compare Eq. 50)

$$+\frac{{\left(\Delta N_{A}\right)}^{2}}{2}\iint \frac{\left(\Delta \rho_{A}^{\left(\text{pol}\right)}\left[\Phi_{B}^{0},r\right]\Delta f_{B}\left(r^{\prime }\right)+\Delta \rho_{B}^{\left(\text{pol}\right)}\left[\Phi_{A}^{0},r\right]\Delta f_{A}\left(r^{\prime }\right)\right)}{\left|r-r^{\prime }\right|}drdr^{\prime }$$ (53)

reagents. There is also a coupling to the first-order electron transfer (Fukui function) term,

$$+{\left(\Delta N_{A}\right)}^{3}\iint \frac{f_{A}\left(r\right)\Delta f_{B}\left(r^{\prime }\right)-f_{B}\left(r\right)\Delta f_{A}\left(r^{\prime }\right)}{\left|r-r^{\prime }\right|}drdr^{\prime }$$ (54)

If *any* of these terms are large, it indicates that the electrostatic potential, polarization, or first-order electron-transfer (Fukui function) terms are likely to be dominant. The dual descriptor is also important when describing the asymptotic behavior of the quadratic and cubic density response functions; it has been argued that the dual descriptor’s chemical relevance may be mostly attributed to those nonlinear polarization terms (Cardenas et al., 2009b).

#### 3.3.6 Energy Change From Electron Transfer; Chemical Potential and Hardness

The remaining terms in Eq. 33 represent the change in energy of the isolated reagents. First of all, the isolated reagents change in energy because the number of electrons in the reagents changes. This leads to the term

$$\Delta N_{A}\left(\mu_{A}^{0}-\mu_{B}^{0}\right)+\frac{{\left(\Delta N_{A}\right)}^{2}}{2}\left(\eta_{A}^{0}+\eta_{B}^{0}\right)$$ (55)

The electronic chemical potential is minus one times the electronegativity, (Parr et al., 1978) and measures the intrinsic strength of a Lewis acid or base (Ayers et al., 2006). The chemical hardness is the key to describing the HSAB principle (Parr and Pearson, 1983). The electron-transfer energy expression in Eq. 55 suffices, all by itself, to derive the HSAB principle (Chattaraj et al., 1991; Ayers, 2005; Chattaraj and Ayers, 2005; Ayers and Cardenas, 2013; Cardenas and Ayers, 2013).

#### 3.3.7 Promotion Energy

Polarizing the reagents so that they can form more favorable electrostatic interactions requires mixing excited-state character into the ground-state fragment densities. This raises the electronic energy of the fragments by,

$$-E_{A}^{\left(\text{pol}\right)}\left[\Phi_{B}^{0}\right]-E_{B}^{\left(\text{pol}\right)}\left[\Phi_{A}^{0}\right]$$ (56)

though this term is doubly-cancelled out (cf. Eq. 46) by the favorable electrostatic contacts that are created (Ayers, 2007a).

#### 3.3.8 Condensed Reactivity Indicators

It is often convenient to replace the pointwise interactions between reactants in the interaction energy model with interactions between the atoms in the reactants. This gives an atom-condensed version of the reaction energy expression (Yang and Mortier, 1986) which strongly resembles the structure of electronegativity equalization molecular mechanics force fields. Letting $w_{a}\left(r\right)$ denote the atomic weight function associated with the *α* ^th^ atom in reactant A, and let $Z_{a}$ and $R_{a}$ denote its nuclear charge and position (consistent with the notation in Eq. 3). The expressions for the atom-condensed charges, condensed Fukui function, (Fuentealba et al., 2016) condensed dual descriptor, and condensed polarization density are, respectively

$$q_{\alpha }=Z_{\alpha }-\int w_{\alpha }\left(r\right)\rho_{A}\left(r\right)dr$$ (57)

$$f_{\alpha }=\int w_{\alpha }\left(r\right)f_{A}\left(r\right)dr$$ (58)

$$\Delta f_{\alpha }=\int w_{\alpha }\left(r\right)\Delta f_{A}\left(r\right)dr$$ (59)

$$\Delta n_{\alpha }=\sum_{\beta \in B}\iint w_{\alpha }\left(r\right){\left(\frac{\delta \rho_{A}\left(r\right)}{\delta v\left(r^{\prime }\right)}\right)}_{N_{A}^{0}}\frac{q_{\beta }}{\left|r^{\prime }-R_{\beta }\right|}drdr^{\prime }.$$ (60)

Here we have elected to use the fragment-of-molecular response method, though the response-of-molecular-fragment method could be used instead (Fuentealba et al., 2000; Ayers et al., 2002; Bultinck et al., 2007; Padmanabhan et al., 2007; Zielinski et al., 2012; Miranda-Quintana R. A., 2016). We could also include dipole (and higher-order multipole) components of the descriptors in Eq. 57, but this would complicate the formula for the polarization energy

$$E_{A}^{\left(\text{pol}\right)}=-\frac{1}{2}\sum_{\alpha \in A}\sum_{\beta \in B}\frac{\Delta n_{\alpha }\cdot q_{\beta }}{\left|R_{\alpha }-R_{\beta }\right|}$$ (61)

and the interaction energy

$$\begin{array}{c} \Delta U_{AB}\left[\Delta N_{A}\right]=\left(T_{s}^{\text{non}-\text{add}}\left[\rho_{A}^{0},\rho_{B}^{0}\right]+E_{xc}^{\text{non}-\text{add}}\left[\rho_{A}^{0},\rho_{B}^{0}\right]\right)\\ +\left(\begin{array}{c} \sum_{\alpha \in A}\sum_{\beta \in B}\frac{q_{\alpha }q_{\beta }}{\left|R_{\alpha }-R_{\beta }\right|}+2\left(E_{A}^{\left(\text{pol}\right)}+E_{B}^{\left(\text{pol}\right)}\right)+\sum_{\alpha \in A}\sum_{\beta \in B}\frac{\Delta n_{\alpha }\Delta n_{\beta }}{\left|R_{\alpha }-R_{\beta }\right|}\\ -\Delta N_{A}\sum_{\alpha \in A}\sum_{\beta \in B}\frac{f_{\alpha }q_{\beta }-q_{\alpha }f_{\beta }}{\left|R_{\alpha }-R_{\beta }\right|}+\Delta N_{A}\sum_{\alpha \in A}\sum_{\beta \in B}\frac{f_{\alpha }\Delta n_{\beta }-\Delta n_{\alpha }f_{\beta }}{\left|R_{\alpha }-R_{\beta }\right|}\\ -{\left(\Delta N_{A}\right)}^{2}\sum_{\alpha \in A}\sum_{\beta \in B}\frac{f_{\alpha }f_{\beta }}{\left|R_{\alpha }-R_{\beta }\right|}-\frac{{\left(\Delta N_{A}\right)}^{2}}{2}\sum_{\alpha \in A}\sum_{\beta \in B}\frac{\Delta f_{\alpha }q_{\beta }+q_{\alpha }\Delta f_{\beta }}{\left|R_{\alpha }-R_{\beta }\right|}\\ +\frac{{\left(\Delta N_{A}\right)}^{2}}{2}\sum_{\alpha \in A}\sum_{\beta \in B}\frac{\Delta f_{\alpha }\Delta n_{\beta }+\Delta n_{\alpha }\Delta f_{\beta }}{\left|R_{\alpha }-R_{\beta }\right|}\\ +{\left(\Delta N_{A}\right)}^{3}\sum_{\alpha \in A}\sum_{\beta \in B}\frac{f_{\alpha }\Delta f_{\beta }-\Delta f_{\alpha }f_{\beta }}{\left|R_{\alpha }-R_{\beta }\right|}+\frac{{\left(\Delta N_{A}\right)}^{4}}{4}\sum_{\alpha \in A}\sum_{\beta \in B}\frac{\Delta f_{\alpha }\Delta f_{\beta }}{\left|R_{\alpha }-R_{\beta }\right|} \end{array}\right)\\ +\left(\Delta N_{A}\left(\mu_{A}^{0}-\mu_{B}^{0}\right)+\frac{{\left(\Delta N_{A}\right)}^{2}}{2}\left(\eta_{A}^{0}+\eta_{B}^{0}\right)-E_{A}^{\left(\text{pol}\right)}-E_{B}^{\left(\text{pol}\right)}\right) \end{array}$$ (62)

This equation is more convenient as a “working formula” than Eq. 33 because it removes the need to perform six-dimensional integrals. We note, however, that once one chooses a suitable energy model, (Parr and Bartolotti, 1982; Parr and Pearson, 1983; Noorizadeh and Shakerzadeh, 2008; Fuentealba and Cardenas, 2013; Heidar-Zadeh et al., 2016a; Miranda-Quintana R. A. and Ayers P. W., 2016) all the integrations in Eq. 33 can be performed analytically by combining the basis-set expansion of one-electron density matrices and the linear response function with the appropriate one- and two-electron integrals.

## 4 Summary

In the density functional theory approach to chemical reactivity (DFT-CR), it is traditional to focus on the reactivity indicators of a single reagent. This is not always adequate, as interactions between reagents can be decisive when, for example, partner-specific interactions are critically important for determining reactivity preferences. In this work, we have derived mathematical formulas for the interaction energy between reagents in terms of chemical reactivity indicators (Eqs. 16, 33) and shown how these expressions are related to existing computational approaches (density-functional embedding, density-functional energy decomposition analysis) and previous reactivity rules (Section 3.2). We also showed that all of the commonly accepted chemical interactions that influence chemical reactivity are contained in the key Eq. 33 (Section 3.3). We believe that this work provides not only a basis for future work in DFT-CR, but that we can rationalize existing DFT-CR success stories by noting that whenever one term in Eq. 33 is decisive, the conventional one-reagent-one-reactivity-indicator approach is likely to be successful. In further contributions in this series we will explore how the two-reagent picture can be used to provide further analytical arguments in favor of several reactivity principles.

## Data Availability

The original contributions presented in the study are included in the article, further inquiries can be directed to the corresponding authors.
